# Treatment response and several patient-reported outcomes are early determinants of future self-efficacy in rheumatoid arthritis

**DOI:** 10.1186/s13075-021-02651-3

**Published:** 2021-10-27

**Authors:** Doumen Michaël, De Cock Diederik, Pazmino Sofia, Bertrand Delphine, Joly Johan, Westhovens René, Verschueren Patrick

**Affiliations:** 1 Department of Development and Regeneration, KU Leuven, Skeletal Biology and Engineering Research Centre, ON IV Herestraat 49 - bus 805, 3000 Leuven, Belgium; 2 Rheumatology, University Hospitals Leuven, Leuven, Belgium

**Keywords:** Rheumatoid arthritis, Self-efficacy, Patient-reported outcomes, Window of opportunity, Self-management, Psychosocial factors, Illness perceptions, Coping, Predictors

## Abstract

**Background:**

Self-efficacy, or patients’ confidence in their ability to control disease and its consequences, was recently prioritised in EULAR recommendations for inflammatory arthritis self-management strategies. However, it remains unclear which factors influence self-efficacy in early rheumatoid arthritis (RA).

**Methods:**

Data were analysed from the 2-year RCT Care in early RA (CareRA), which studied remission-induction treatment regimens for early RA. Participants completed the Arthritis Self-Efficacy Scale (ASES), Short-Form 36 (SF-36), Revised Illness Perception Questionnaire (IPQ-R), Utrecht Coping List (UCL), RAQoL and Health Assessment Questionnaire (HAQ). Depending on time to first remission (DAS28-CRP < 2.6) and persistence of remission, treatment response was defined as persistent response, secondary failure, delayed response, late response or non-response. The association between ASES scores and clinical/psychosocial factors was explored with Spearman correlation and multivariate linear mixed models. Baseline predictors of week 104 ASES were identified with exploratory linear regression followed by multiple regression of significant predictors adjusted for DAS28-CRP, HAQ, treatment arm, treatment response, cumulative CRP/SJC28 and demographic/serologic confounders.

**Results:**

All 379 patients had a recent diagnosis of RA and were DMARD-naïve at study initiation. Most patients were women (69%) and RF/ACPA-positive (66%), and the mean (*SD*) age was 52 (13) years. For all tested outcome measures, better perceived health correlated with higher self-efficacy. While patient-reported factors (HAQ, SF-36, RAQoL, IPQ-R, pain, fatigue and patient’s global assessment) showed moderate/strong correlations with ASES scores, correlations with physician-reported factors (physician’s global assessment, SJC28), TJC28 and DAS28-CRP were weak.

Only more favourable outcomes on patient-reported factors and DAS28-CRP were associated with higher ASES scores at each time point.

An earlier, persistent treatment response predicted higher ASES scores at both weeks 52 and 104. Significant baseline predictors of week 104 ASES included HAQ; SF-36 mental component score, vitality, mental health and role emotional; IPQ-R illness coherence, treatment control, emotional representations and consequences; UCL Passive reacting; and the RAQoL.

**Conclusions:**

Patient-reported outcomes and treatment response were early determinants of long-term self-efficacy in an early RA trial. These results provide further relevance for the window of opportunity in an early treat-to-target strategy and could help to timely identify patients who might benefit from self-management interventions.

**Trial registration:**

EudraCT 2008-007225-39

**Supplementary Information:**

The online version contains supplementary material available at 10.1186/s13075-021-02651-3.

## Background

Rheumatoid arthritis (RA) is the most common form of chronic inflammatory arthritis, and it considerably impacts patients’ daily functioning and quality of life [1]. Although remission of RA has become a realistic treatment target, around one-in-five patients still report ongoing symptoms even when the disease appears clinically well-controlled [2, 3]. Since adequate control of ongoing symptoms like pain and fatigue is a crucial goal for patients, it is essential not only to induce remission by early and intensive treatment, but also to timely address these unmet patient needs [4]. In addition to pain and fatigue, the unmet needs of patients with RA often include psychosocial factors such as the impact on mental health and social functioning [5]. Recently, there has been an increasing awareness that these psychosocial aspects have a significant influence on patients’ perceived symptoms and quality of life [6–8].

As in every chronic disease, an important long-term aspect in the care for patients with RA is self-efficacy, defined as the confidence in one’s ability to carry out a task with the desired outcome [9]. For patients with RA, self-efficacy mainly relates to confidence in their ability to better control the disease and its consequences with actions they can take themselves. Self-efficacy is therefore a major contributor to self-management behaviour and active coping, which in turn are promoters of psychological adjustment to chronic illness [10]. In other words, patients who are more self-efficacious can be more actively involved in the management of their disease, in a process of shared decision-making [11]. Shared decision-making involves patients working together with rheumatologists to provide the best possible care with regard to both scientific evidence and patient-specific goals and preferences [12]. In recent years, shared decision-making and disease self-management have gained increasing attention, both in usual care and as the cornerstone of innovative care models like patient-initiated consultations and mobile health (mHealth)-guided follow-up [13]. Consequently, self-efficacy was identified as a priority in the recent EULAR recommendations for the implementation of self-management strategies for inflammatory arthritis [14]. Moreover, with a view to improved shared decision-making, numerous intervention studies have shown that self-efficacy beliefs are not static personality traits but can be improved with personalised patient education and psychological support [15–17].

Therefore, it is important to gain a clear understanding of which factors influence self-efficacy in RA. Stronger self-efficacy beliefs have been associated with lower levels of pain, fatigue, physical disability and emotional distress [18]. Moreover, a negative association between disease severity and self-efficacy has previously been reported [19]. Finally, feeling more self-efficacious seems to be related to psychosocial wellbeing in patients with early RA [20].

However, the studies reporting these associations are characterised by considerable heterogeneity in outcome measures, and most of those studies have been limited to describing cross-sectional correlations [21]. Moreover, few studies have been able to identify predictors of self-efficacy in patients with RA, and the predictors that have been suggested have often not been confirmed in other publications [21–23].

Therefore, we aimed to investigate which disease-related and psychosocial factors are associated with self-efficacy across time in patients with RA. Moreover, we looked specifically at the early stages of disease, where effective interventions might have the largest impact.

## Methods

### Study design and participants

This study was an observational post hoc analysis of the Care in early RA (CareRA) trial. CareRA (EudraCT number: 2008-007225-39) was a prospective, multicentre, 2-year, open-label pragmatic randomised controlled trial comparing different remission-induction treatment regimens for early RA [24]. The trial included 379 patients from 13 Belgian rheumatology centres. All included patients had a recent diagnosis of RA (<1 year ago) and had not received disease-modifying antirheumatic drugs (DMARDs) before study initiation. Details on the study protocol and primary outcomes have been published elsewhere [24, 25]. In short, each treatment arm consisted of methotrexate with or without additional conventional synthetic DMARDs (csDMARDs) and with or without temporary glucocorticoids. The ethics committee of all participating centres approved the study protocol, and all included patients provided written informed consent. For this observational study, which included the entire CareRA cohort, no additional ethical approval was required.

### Assessments and outcomes

The primary aim of this study was to identify clinical and psychosocial factors associated with self-efficacy across time, as well as early predictors of self-efficacy, in patients with early RA. Self-efficacy was measured by the 2-subscale version of the Arthritis Self-Efficacy Scale (ASES) at week 52 and week 104 [26]. The pain (PSE) and other symptoms (OSE) subscales measure the patient’s perceived confidence in their ability to control arthritis pain and to control symptoms of arthritis other than pain, respectively, with other means than medication. PSE and OSE were scored separately and summed to derive a total ASES score. Scores on both the PSE and OSE subscales range from 1 to 10 (“very uncertain” to “very certain”). The total ASES score thus ranges from 2 to 20, with higher scores indicating stronger self-efficacy beliefs.

### Clinical variables

Participants were clinically assessed at screening, baseline and weeks 4, 8, 16, 28, 40, 52, 65, 78, 91 and 104 to collect tender and swollen joint counts (TJC28/SJC28), patient’s and physician’s global assessment of disease activity (PGA/PhGA) on a visual analogue scale (VAS) of 100mm and laboratory markers of inflammation including C-reactive protein (CRP) and erythrocyte sedimentation rate (ESR). Patients’ demographic characteristics were recorded at baseline, including comorbidity burden in the form of the Rheumatic Diseases Comorbidity Index (RDCI) [27]. Disease Activity Score in 28 joints with CRP (DAS28-CRP) was calculated as a composite disease activity measure [28], and DAS28-CRP <2.6 was defined as remission for all analyses [29]. Treatment was initiated at the baseline visit. Treatment response was specified as a categorical variable with 5 levels: (1) persistent response, defined as sustained remission from week 16 to week 104; (2) secondary failure, defined as remission by week 16 with later loss of remission; (3) delayed response, defined as a first remission between week 16 and week 52; (4) late response, defined as a first remission after week 52; and (5) non-response, defined as no remission within 104 weeks.

### Psychosocial variables

In addition to the ASES, numerous other patient-reported outcomes (PROs) were collected at different timepoints. These include, among others, at baseline, weeks 16, 52, and 104: pain and fatigue on a VAS of 100mm, physical function measured by the Health Assessment Questionnaire (HAQ), illness perceptions assessed by the Revised Illness Perception Questionnaire (IPQ-R) and multidimensional quality of life as reflected by the Short-Form 36 health survey (SF-36) and the RAQoL [30–33]. Furthermore, coping strategies were collected at baseline and week 16 using the Utrecht Coping List (UCL) [34].

The IPQ-R measures 9 dimensions of illness perception [31]. Among these, the dimensions of illness coherence, personal control, treatment control, emotional representations and perceived consequences were selected as most relevant to our research question. Higher scores on illness coherence imply a better understanding of the disease, while higher outcomes on personal control and treatment control indicate a higher perceived likelihood of personal interventions or treatment controlling the disease. By contrast, higher scores on emotional representations and consequences represent a stronger perceived impact of the disease’s negative consequences.

The SF-36 measures 8 dimensions of health: 4 physical dimensions (physical function, role physical, bodily pain and general health; compiled into a physical component score (PCS)) and 4 mental dimensions (vitality, social function, role emotional and mental health; compiled into a mental component score (MCS)) [32]. Higher scores on each of the SF-36 dimensions (0–100) imply better patient-perceived health. For this study, we placed particular emphasis on the SF-36 mental components, since the physical dimensions were considered to be well-represented by other included PROs. The RAQoL is an RA-specific quality of life (QoL) instrument consisting of 30 “yes/no” questions, resulting in a score of 0–30 with higher scores indicating lower QoL [33]. Finally, the UCL assesses seven distinct coping strategies, with higher scores corresponding with more use of a certain strategy [34].

### Statistical analysis

Missing PRO data were first handled as outlined in the individual questionnaire manuals. Next, missing data were assumed to be missing at random and imputed with multiple imputation by classification and regression trees (total missingness 16%, *n* = 100 imputations). Analyses were carried out in each imputed dataset and then pooled using Rubin’s rules. Descriptive statistics were reported as means (± *SD*), medians (± *IQR*) or proportions depending on data distribution. Correlations between ASES scores and the selected clinical and psychosocial variables were reported as Spearman coefficients.

To describe the association of the selected clinical and psychosocial variables with total ASES scores across time, linear mixed models (LMMs) with a random intercept were constructed. This method makes optimal use of longitudinal data by considering repeated observations within individual patients, allowing for a different baseline value for each patient. Because ASES was only collected at 2 timepoints, we did not include a random slope for time in these mixed models. All LMMs were multivariate models adjusting for concurrent DAS28-CRP and HAQ, as well as time, treatment arm, age, gender and the presence of rheumatoid factor (RF) or anti-citrullinated peptide antibodies (ACPA) as covariates. When the independent variable of interest was a component of the DAS28-CRP, the other DAS28-CRP components were included as individual covariates instead of DAS28-CRP.

To identify baseline predictors of long-term self-efficacy, we first constructed exploratory, univariate linear regression models using the total ASES score at week 104 as the dependent variable and the selected clinical and psychosocial variables at baseline, including the individual questionnaire dimensions, as predictors. Statistically significant predictors of week 104 ASES (*p* < 0.05) were then included as independent variables in multiple linear regression models adjusted for categorical treatment response, treatment arm, age, gender, the presence of RF or ACPA and baseline HAQ and DAS28-CRP (or its components) as relevant covariates. In addition, these models were also adjusted for the cumulative sum of CRP and SJC28 levels across the 2 years of follow-up, as longitudinal markers of disease-related inflammation. To account for the increased risk of type I error due to multiple comparisons (*n* = 18 prediction models were constructed), a Bonferroni correction was applied, setting the significance level at *α* = 0.003 instead of 0.05 for all analyses following the exploratory step. Finally, a mediation analysis was carried out to gain a better understanding of how treatment response could affect self-efficacy, when considering other treatment-related predictors collected at the same timepoints as the ASES. Confidence intervals for the mediation analysis were obtained through bootstrapping (5000 iterations of random sampling with replacement). All statistical analyses were carried out in R Studio version 1.3.1093, with inclusion of the packages *mice*, *lmerTest*, *lavaan* and *lavaanPlot*.

## Results

### Patient characteristics

A total of 379 patients with early RA were included in CareRA between January 2009 and May 2013, 322 (85%) of whom completed the 2-year study. After imputation of missing data, all 379 patients were included in this post-hoc analysis. All patients had a recent diagnosis of RA and were DMARD-naïve at study initiation, with a mean (*SD*) DAS28-CRP of 4.8 (1.3) at baseline. The majority of patients were women (69%) and RF or ACPA-positive (66%) and the mean (*SD*) age was 52 (13) years. Table 1 presents baseline demographic, clinical and patient-reported outcome characteristics.

**Table 1:** Baseline characteristics of participants included in CareRA (*n* = 379)

	Distribution
**Demographic variables**	
Age, years	52 (13)
BMI, kg/m^2^	26 (4)
Women, *n* (%)	262 (69)
Smokers, *n* smoked ever (%)	209 (55)
RF positive, *n* (%)	252 (66)
ACPA positive, *n* (%)	249 (66)
Erosive disease, *n* (%)	97 (26)
RDCI, mean (*SD*)	0.84 (1.15)
**Clinical variables**	
DAS28-CRP	4.8 (1.3)
TJC28, median (*IQR*)	7 (8)
SJC28	6 (8)
PGA, mm (0–100)	55 (24)
Pain, mm (0–100)	56 (24)
Fatigue, mm (0–100)	48 (24)
PhGA, mm (0–100)	52 (19)
ESR, mm/h, median (*IQR*)	23 (30)
CRP, mg/L, median (*IQR*)	21 (78)
HAQ (0–3), median (*IQR*)	1 (1)

Results are reported as mean (*SD*) unless otherwise specified. *IQR* interquartile range, *BMI* body mass index, *RF* rheumatoid factor, *ACPA* anti-citrullinated peptide antibody, *RDCI* Rheumatic Diseases Comorbidity Index, *DAS28* Disease Activity Score in 28 joints, *CRP* C-reactive protein, *TJC28* tender joint count in 28 joints, *SJC28* swollen joint count in 28 joints, *PGA* patient’s global assessment of disease activity, *PhGA* physician’s global assessment of disease activity, *ESR* erythrocyte sedimentation rate, *HAQ* Health Assessment Questionnaire

### Factors associated with self-efficacy

Table 2 presents coefficients for the correlation between both ASES subscales and the selected clinical and patient-reported variables at all available timepoints. As expected, the ASES PSE and OSE subscales were strongly correlated with each other (*r*_S_ = 0.72) and with the total score (since this is the sum of the two subscales). For all included outcome measures, better perceived health was associated with stronger self-efficacy beliefs. In general, patient-reported variables (HAQ, SF-36 component scores, RAQoL, IPQ-R, pain, fatigue and PGA) showed moderate to strong correlations with OSE, and in most cases, these correlations were notably stronger than those with PSE. By contrast, physician-reported variables (PhGA, SJC28), TJC28 and DAS28-CRP were only weakly correlated with both ASES subscales. Furthermore, laboratory measures of inflammation (CRP and ESR) showed only very weak correlations with ASES scores. None of the included variables showed a substantially stronger correlation with PSE than with OSE.

**Table 2:** Spearman coefficients for the correlation between ASES subscales and clinical and psychosocial variables at all available timepoints

	ASES pain	ASES other symptoms	ASES total
DAS28-CRP	−0.35	−0.33	−0.37
HAQ	−**0.48**	−**0.55**	−**0.55**
Pain (VAS)	−**0.46**	−**0.46**	−**0.50**
Fatigue (VAS)	−**0.45**	−**0.51**	−**0.51**
PGA (VAS)	−**0.46**	−**0.47**	−**0.51**
SF-36 PCS	**0.58**	**0.61**	**0.64**
SF-36 MCS	0.33	**0.52**	**0.44**
IPQ-R illness coherence	0.37	**0.50**	**0.46**
IPQ-R treatment control	**0.40**	**0.46**	**0.46**
IPQ-R personal control	0.29	0.27	0.30
IPQ-R consequences	−**0.58**	−**0.64**	−**0.65**
IPQ-R emotional representations	−**0.46**	−**0.59**	−**0.56**
RAQoL	−**0.57**	−**0.68**	−**0.67**
PhGA (VAS)	−0.32	−0.34	−0.36
TJC28	−0.32	−0.31	−0.34
SJC28	−0.19	−0.17	−0.20
CRP	−0.07	−0.06	−0.07
ESR	−0.07	−0.07	−0.08

*ASES* Arthritis Self-Efficacy Scale, *DAS28* Disease Activity Score in 28 joints, *HAQ* Health Assessment Questionnaire, *VAS* visual analogue scale, *PGA* patient’s global assessment of disease activity, *SF-36* Short-Form 36, PCS physical component score, *MCS* mental component score, *IPQ-R* Revised Illness Perception Questionnaire, *PhGA* physician’s global assessment of disease activity, *TJC28* tender joint count in 28 joints, *SJC28* swollen joint count in 28 joints, *CRP* C-reactive protein, *ESR* erythrocyte sedimentation rate

In the multivariate longitudinal models, only better scores on patient-reported variables and DAS28-CRP were significantly associated with higher total ASES scores across time, which was not the case for physician-reported and laboratory measures of disease activity (Table 3). In the case of DAS28-CRP and RAQoL, these associations interacted with HAQ: more specifically, the additional effect of DAS28-CRP or RAQoL on the total ASES score decreased with increasing HAQ scores. A combination of DAS28-CRP, HAQ, age, gender, treatment arm, auto-antibody status and different individual PROs explained 60–69% of the total variance in the total ASES score across time.

**Table 3:** Variables associated with total ASES score across time, based on multivariate linear mixed models

Variable	Coefficient (***95% CI***)	***p***-value^**⊥**^	Conditional ***R***^**2**^	Interactions
DAS28-CRP	−0.22 (−0.32, −0.11)	**<0.001**	0.614	HAQ*
HAQ	−1.06 (−1.27, −0.84)	**<0.001**	0.610	-
Pain (VAS)	−0.14 (−0.19, −0.09)	**<0.001**	0.603	-
Fatigue (VAS)	−0.15 (−0.19, −0.10)	**<0.001**	0.606	-
PGA (VAS)	−0.16 (−0.21, −0.11)	**<0.001**	0.594	-
SF-36 MCS	0.40 (0.29, 0.50)	**<0.001**	0.607	-
IPQ-R illness coherence	0.12 (0.09, 0.15)	**<0.001**	0.617	-
IPQ-R treatment control	0.16 (0.12, 0.20)	**<0.001**	0.619	-
IPQ-R personal control	0.09 (0.06, 0.11)	**<0.001**	0.608	-
IPQ-R consequences	−0.17 (−0.19, −0.15)	**<0.001**	0.690	-
IPQ-R emotional representations	−0.11 (−0.12, −0.09)	**<0.001**	0.634	-
RAQoL	−0.11 (−0.12, −0.09)	**<0.001**	0.640	HAQ**
PhGA (VAS)	−0.12 (−0.19, −0.04)	**0.002**	0.613	-
TJC28	−0.01 (−0.06, 0.03)	0.590	0.594	-
SJC28	0.01 (−0.04, 0.07)	0.630	0.594	-
CRP	0.00 (−0.01, 0.01)	0.550	0.594	-
ESR	0.00 (0.00, 0.01)	0.320	0.597	-

^**⊥**^Bonferroni correction for multiple comparisons was applied, setting the significance level at *p* < 0.003

*Interaction (*ß* = 0.24; *95% CI* = 0.12–0.36; *p* < 0.001) implies that the additional effect of DAS28-CRP on self-efficacy (SE) decreases with increasing HAQ

**Interaction (*ß* = 0.05; *95% CI* = 0.03–0.08; *p* < 0.001) implies that the additional effect of RAQoL on SE decreases with increasing HAQ

*ASES* Arthritis Self-Efficacy Scale, *DAS28* Disease Activity Score in 28 joints, *HAQ* Health Assessment Questionnaire, *VAS* visual analogue scale, *PGA* patient’s global assessment of disease activity, *SF-36 MCS* Short-Form 36 mental component score, *IPQ-R* Revised Illness Perception Questionnaire, *PhGA* physician’s global assessment of disease activity, *TJC28* tender joint count in 28 joints, *SJC28* swollen joint count in 28 joints, *CRP* C-reactive protein, *ESR* erythrocyte sedimentation rate

### Predictors of self-efficacy

**Fig. 1:**
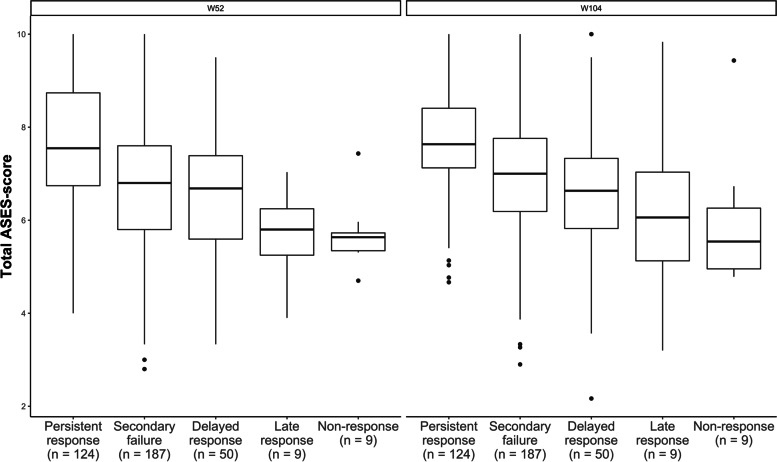
Total ASES score at week 52 and week 104, stratified by treatment response profile. Response to treatment was associated with total scores on the Arthritis Self-Efficacy Scale (ASES) at week 52 and week 104, with more favourable responses to treatment resulting in stronger self-efficacy beliefs after both 1 and 2 years of treatment

**Fig. 2:**
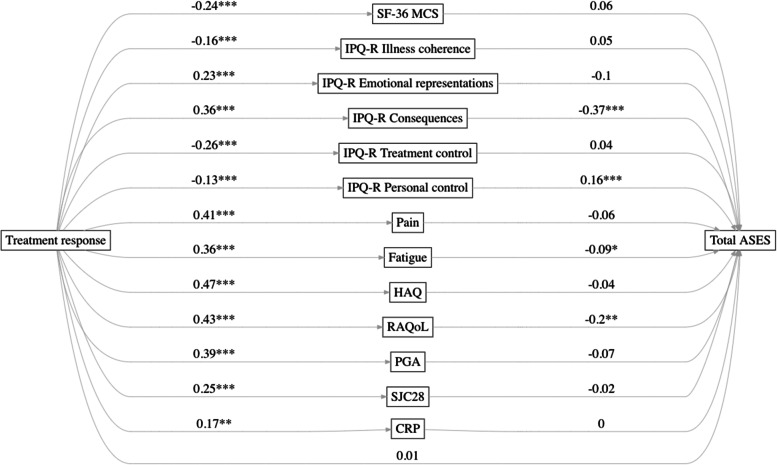
Mediators of the association between treatment response and total ASES score at week 104. Reported are the standardised regression coefficients with indicators of significance (*p < 0.05; **p < 0.01; ***p < 0.001). Treatment response was treated as an ordinal variable with lower values representing earlier and more persistent response. *ASES* Arthritis Self-Efficacy Scale, *SF-36* Short-Form 36, *MCS* mental component score, *IPQ-R* Revised Illness Perception Questionnaire, *HAQ* Health Assessment Questionnaire, *PGA* patient’s global assessment of disease activity, *SJC28* swollen joint count in 28 joints, *CRP* C-reactive protein

Patients who achieved a more favourable treatment response had higher total ASES scores at week 104 (Fig. 1, Table 4). The highest ASES scores were seen in the persistent response group, followed by the groups with secondary failure, delayed response, late response and non-response, respectively. In the multivariate prediction models, after adjusting for baseline HAQ and DAS28-CRP, treatment arm, auto-antibody status, cumulative inflammation and demographics, treatment response remained a significant predictor of total ASES scores at week 104 (Table 5). In a mediation analysis, the effect of treatment response on later self-efficacy was mediated by IPQ-R dimensions consequences and personal control, the RAQoL and VAS for fatigue, while there was no significant mediation by CRP, SJC28, PGA, HAQ, VAS for pain, SF-36 MCS or other IPQ-R dimensions (Fig. 2, Supplement 1). Although there was a significant total effect, the direct effect of treatment response on later ASES scores was not significant after adjusting for these mediators.

**Table 4:** Baseline and treatment-related predictors of total ASES score at week 104, based on univariate models

Variable	Coefficient (***95% CI***)	***p***-value	Variable	Coefficient	***p***-value
**Clinical/demographic**			**IPQ-R**		
Age	0.01 (0.00, 0.02)	0.056	Illness coherence	0.09 (0.06, 0.13)	**<0.001**
Gender (male)	0.36 (0.06, 0.66)	**0.021**	Treatment control	0.11 (0.05, 0.17)	**<0.001**
Ab-status (+)	−0.03 (−0.37, 0.31)	0.856	Personal control	0.08 (0.04, 0.12)	**<0.001**
Erosive disease (+)	0.14 (−0.19, 0.46)	0.409	Emotional repr.	−0.09 (−0.11, −0.07)	**<0.001**
RDCI	−0.05 (−0.17, 0.07)	0.442	Consequences	−0.09 (−0.13, −0.06)	**<0.001**
Treatment type	0.00 (−0.11, 0.10)	0.981	**UCL**		
Treatment response	−0.51 (−0.66, −0.36)	**<0.001**	Seeking social support	0.04 (0.00, 0.08)	0.065
HAQ	−0.57 (−0.76, −0.37)	**<0.001**	Passive reacting	−0.11 (−0.15, −0.06)	**<0.001**
DAS28-CRP	−0.07 (−0.18, 0.04)	0.222	Active tackling	0.05 (0.01, 0.09)	**0.028**
PGA (VAS)	−0.13 (−0.19, −0.07)	**<0.001**	Palliative reacting	−0.02 (−0.06, 0.02)	0.364
TJC28	−0.01 (−0.03, 0.02)	0.543	Avoidance	−.04 (−0.08, 0.01)	0.093
SJC28	0.01 (−0.01, 0.04)	0.356	Expr. of emotion	−0.03 (−0.11, 0.05)	0.484
CRP	0.00 (−0.01, 0.00)	0.108	Reassuring thoughts	0.02 (−0.04, 0.08)	0.504
ESR	0.00 (−0.01, 0.01)	0.782	**SF-36**		
PhGA (VAS)	−0.09 (−0.16, −0.02)	**0.021**	MCS	0.41 (0.29, 0.53)	**<0.001**
Pain (VAS)	−0.12 (−0.18, −0.06)	**<0.001**	VT	0.25 (0.19, 0.32)	**<0.001**
Fatigue (VAS)	−0.14 (−0.20, −0.09)	**<0.001**	SF	0.16 (0.11, 0.21)	**<0.001**
**RAQoL**			MH	0.27 (0.20, 0.34)	**<0.001**
RAQoL	−0.07 (−0.09, −0.05)	**<0.001**	RE	0.09 (0.06, 0.12)	**<0.001**

Results were obtained from univariate linear regression models predicting the total ASES score at week 104. For this exploratory stage, no correction for multiple comparisons was applied

*ASES* Arthritis Self-Efficacy Scale, *RDCI* Rheumatic Diseases Comorbidity Index, *HAQ* Health Assessment Questionnaire, *DAS28-CRP* Disease Activity Score in 28 joints with C-reactive protein, *PGA* patient’s global assessment of disease activity, *TJC28* tender joint count in 28 joints, *SJC28* swollen joint count in 28 joints, *CRP* C-reactive protein, *ESR* erythrocyte sedimentation rate, *PhGA* physician’s global assessment of disease activity, *VAS* visual analogue scale, *SF-36* Short-Form 36, *MCS* mental component score, *VT* vitality, *SF* social function, *MH* mental health, *RE* Role Emotional, *IPQ-R* Revised Illness Perception Questionnaire, *UCL* Utrecht Coping List

**Table 5:** Baseline and treatment-related predictors of the total ASES score at week 104, based on multivariate models

Variables	Coefficient (***95% CI***)	***p***-value^**⊥**^	Adjusted ***R***^**2**^
**Clinical/demographic**			
Gender (male)	0.32 (0.03, 0.60)	0.030	0.178
Treatment response	−0.46 (−0.65, −0.27)	**<0.001**	0.178
HAQ	−0.73 (−0.97, −0.49)	**<0.001**	0.178
Pain (VAS)	−0.04 (−0.11, 0.03)	0.316	0.177
Fatigue (VAS)	−0.06 (−0.12, 0.01)	0.083	0.182
PGA	−0.01 (−0.08, 0.07)	0.733	0.182
PhGA	−0.01 (−0.11, 0.09)	0.607	0.175
**SF-36**			
MCS	0.31 (0.19, 0.43)	**<0.001**	0.230
Vitality	0.16 (0.08, 0.24)	**<0.001**	0.210
Social function	0.08 (0.02, 0.15)	0.008	0.191
Mental health	0.21 (0.13, 0.28)	**<0.001**	0.235
Role emotional	0.07 (0.04, 0.10)	**<0.001**	0.218
**IPQ-R**			
Illness coherence	0.07 (0.04, 0.10)	**<0.001**	0.215
Treatment control	0.09 (0.03, 0.14)	**0.002**	0.197
Personal control	0.05 (0.01, 0.09)	0.008	0.191
Emotional representations	−0.07 (−0.10, −0.05)	**<0.001**	0.261
Consequences	−0.07 (−0.10, −0.03)	**<0.001**	0.208
**UCL**			
Passive reacting	−0.08 (−0.12, −0.04)	**<0.001**	0.186
Active tackling	0.03 (−0.01, 0.07)	0.090	0.182
**RAQoL**	−0.04 (−0.07, −0.02)	**0.001**	0.199

Results were obtained from multivariate linear regression models predicting the total ASES score at week 104 by predictors that were significant in univariate analyses and adjusting for age, gender, treatment type and response, auto-antibody status, cumulative CRP, cumulative SJC28 and baseline HAQ and DAS28-CRP or its components. Treatment response was treated as an ordinal variable with lower values representing earlier and more persistent response

^**⊥**^Bonferroni correction for multiple comparisons was applied, setting the significance level at *p* < 0.003

*ASES* Arthritis Self-Efficacy Scale, *SF-36* Short-Form 36, *MCS* mental component score, *IPQ-R* Revised Illness Perception Questionnaire, *UCL* Utrecht Coping List

In addition to treatment response, better perceived health status at baseline for the following PROs was independently associated with higher ASES scores at week 104 in both univariate and multivariate models: HAQ (*ß* = −0.73, *p* < 0.001); SF-36-dimensions MCS (*ß* = 0.31, *p* < 0.001), vitality (*ß* = 0.16, *p* < 0.001), mental health (*ß* = 0.21, *p* < 0.001) and role emotional (*ß* = 0.07, *p* < 0.001); IPQ-R-dimensions illness coherence (*ß* = 0.07, *p* < 0.001), treatment control (*ß* = 0.09, *p* = 0.003), emotional representations (*ß* = −0.07, *p* < 0.001) and consequences (*ß* = −0.07, *p* < 0.001); the UCL-dimension of Passive reacting (*ß* = −0.08, *p* < 0.001); and the RAQoL (*ß* = −0.04, *p* = 0.001).

However, the predictive effects of gender; baseline VAS for PGA, PhGA, pain or fatigue; the SF-36-dimension social function; the IPQ-R-dimension Personal control and the UCL-dimension Active tackling were no longer significant after adjusting for multiple comparisons and covariates (Tables 4 and 5).

## Discussion

In our study, the self-efficacy beliefs of patients with early RA were more strongly associated with the response to treatment and with patient-reported outcomes—including pain, fatigue, physical function, mental health, illness perceptions and quality of life—than with physician-reported and laboratory measures of disease activity. Furthermore, our results suggest that psychosocial factors, even early in the disease course, can predict long-term self-efficacy beliefs independently of treatment response or objective measures of disease activity.

Of particular interest is our finding that a better response to treatment appears to be an independent contributor to stronger long-term self-efficacy beliefs in patients with early RA. Current treatment guidelines for RA recommend starting treatment early and intensively, aiming to achieve remission or at least low disease activity within a certain “window of opportunity” [35, 36]. The importance of the window of opportunity has been further emphasised by previous research showing that early achievement of treatment targets also results in better psychosocial outcomes [37]. Our findings illustrate that this appears to be equally the case for self-efficacy beliefs, with patients who demonstrated an early and persistent treatment response scoring highest on the ASES after both 1 and 2 years of treatment.

Interestingly, although we did find a correlation between measures of disease activity and self-efficacy at both available timepoints, disease activity and symptoms like pain and fatigue at baseline were not predictive of future self-efficacy. Moreover, the association of early and persistent treatment response with later self-efficacy remained robust even after adjusting for cumulative markers of inflammation. Finally, the results of our mediation analysis suggest that the effect of early and persistent treatment response on self-efficacy is mainly indirect. Treatment response seems to exert its influence primarily through improvements in various psychosocial outcomes and illness perceptions, but not through improvements in pain, physical function, inflammation or swollen joint counts. These findings illustrate that the experience of achieving a satisfactory response to treatment seemingly plays a larger role in building self-management confidence than through better control of disease activity or inflammation as such.

In addition, our results show moderate to strong correlations between several PROs and self-efficacy. These findings are in line with those of a recent meta-analysis, which demonstrated significant associations between the ASES and key areas of functioning in patients with RA or osteoarthritis [18]. However, the associations reported by studies included in this meta-analysis were rather heterogeneous, and most of these studies did not investigate outcome measures relating to illness perceptions or coping. Remarkably, only few studies have investigated associations between self-efficacy and psychological factors beyond QoL or depression/anxiety, but these studies have mostly shown a relation between higher self-efficacy and positive affect, acceptance of illness or active coping [38, 39]. Our findings similarly suggest that optimal illness coherence, more positive illness perceptions and less emotional impact of disease are associated with stronger self-efficacy beliefs.

Furthermore, the ASES subscale measuring self-efficacy for symptoms other than pain (OSE) showed markedly stronger correlations with most patient-reported measures of health and QoL, including fatigue, when compared to the pain subscale (PSE). Only for pain, DAS28-CRP and PGA were correlations with OSE and PSE comparable. These findings suggest that experiencing adequate control of pain and disease activity equally influences patients’ confidence in their ability to manage pain or other symptoms, whereas self-efficacy for symptoms other than pain appears to depend more on QoL in a broader psychosocial sense. This difference between ASES subscales has been reported before and further underlines that managing pain and other symptoms, like fatigue, are perceived by patients with RA as different challenges, which in turn are influenced by different factors [18, 21].

It is notable that previous research has identified physical function as one of the factors strongly associated with self-efficacy beliefs [40]. Our findings confirm this association but suggest a more complex interplay between both concepts. For instance, in the longitudinal models, the association between DAS28-CRP and ASES scores across time interacted with HAQ, with the additional effect of DAS28-CRP on self-efficacy decreasing with increasing HAQ. Thus, the association between disease activity and self-efficacy appears to become less important when the patient experiences a heavier burden of physical disability. Interestingly, analogous results were obtained for the association between QoL (measured by the RAQoL) and self-efficacy. It is important to emphasise that self-efficacy represents one’s confidence, rather than one’s ability, to successfully perform a task [9]. In other words, our results suggest that when a patient’s physical function is heavily impaired, it is the patient’s perceived lack of ability to function that primarily affects their confidence to self-manage.

Finally, our study identified a number of early predictors of long-term self-efficacy beliefs in patients with RA. Interestingly, psychosocial factors were the strongest early predictors of future self-efficacy, rather than perceived pain, fatigue or classic measures of disease activity such as joint counts or biochemical inflammation. For instance, better perceived mental health and QoL, more positive illness perceptions and a less passive coping style before treatment initiation were associated with stronger self-efficacy beliefs after 2 years of treatment. These results are in line with previous research that has shown an important influence of psychological health on self-efficacy or vice versa [23, 41]. However, it is particularly noteworthy that patients’ self-efficacy beliefs following treatment appear to be influenced by psychological wellbeing, coping and illness perceptions as early as the time of treatment initiation. These results suggest that patients with certain psychosocial characteristics, such as a passive coping style or a stronger emotional impact of disease, are at risk to maintain a lower confidence in their ability to manage their disease, even when treatment has proved effective. Since confidence to self-manage is crucial to facilitate treatment strategies that actively involve and empower the patient [14], our findings further underline the importance of a person-centred, holistic treatment approach to complement pharmacological treatment, even early in the disease course. A number of studies have already shown that patients’ self-efficacy beliefs can be improved by needs-based patient education and psychological interventions [17, 42]. Our study provides further evidence that early attention to specific psychosocial risk factors might help to identify those patients that could benefit the most from these interventions.

Our study has some limitations. First, not all PROs were collected at every visit. For instance, coping mechanisms were only assessed at baseline and week 16, while the ASES was collected at week 52 and week 104. Therefore, a cross-sectional correlation between coping and self-efficacy could not be obtained. Furthermore, because the ASES was not collected at baseline, conclusions could not be made about changes in self-efficacy after treatment or the effect of psychosocial predictors on changes in self-efficacy from baseline. However, baseline ASES scores would be less relevant to our research question, since it could be argued that self-efficacy needs time to develop, and patients likely cannot be expected to feel self-efficacious before treatment has even been initiated.

Second, coping was assessed by the Utrecht Coping List. Although the UCL is a validated instrument to measure coping, it has not yet been validated within a population of patients with RA. Therefore, it is possible that the choice of psychometric instrument had an additional influence on the obtained results.

Third, this study is an exploratory post-hoc analysis of data from a trial on pharmacological treatment strategies, rather than the result of a psychological or educational intervention. Therefore, we cannot draw conclusions about the possible causal nature of the reported associations.

However, our study does provide a unique insight into the relationship between psychosocial wellbeing and self-efficacy. For instance, our assessment of psychosocial wellbeing is based on various PROs relating to mental health, social functioning, illness perceptions and coping strategies. Moreover, self-efficacy was measured by a validated instrument at 2 different timepoints during the early disease course.

## Conclusions

In conclusion, our findings suggest a possible role for psychosocial characteristics and the response to treatment as early determinants of the long-term self-efficacy beliefs of patients with RA. These results could be seen as further evidence on the importance of the window of opportunity in an early treat-to-target strategy. In addition, our findings suggest that attention to psychosocial factors could help to timely identify patients who might benefit the most from interventions to improve self-efficacy, or to identify the optimal target audience for treatment strategies that rely heavily on patient self-management. However, further research is needed to confirm these findings in the context of a prospective interventional trial aiming to improve self-efficacy.

## Supplementary Information

**Additional file 1: Supplement 1**. Standardised coefficients and bootstrapped CIs for direct and indirect effects of treatment response on self-efficacy.

## Data Availability

The datasets used and/or analysed during the current study are available from the corresponding author on reasonable request.

## Abbreviations

RA: Rheumatoid arthritis
mHealth: Mobile health
EULAR: European League Against Rheumatism
ASES: Arthritis Self-Efficacy Scale
PSE: Pain self-efficacy
OSE: Other symptoms self-efficacy
CareRA: Care in early RA
DMARD: Disease-modifying antirheumatic drug
csDMARD: Conventional synthetic disease-modifying antirheumatic drug
TJC28: Tender joint count in 28 joints
SJC28: Swollen joint count in 28 joints
PGA: Patient’s global assessment of disease activity
PhGA: Physician’s global assessment of disease activity
VAS: Visual analogue scale
CRP: C-reactive protein
ESR: Erythrocyte sedimentation rate
DAS28-CRP: Disease Activity Score in 28 joints with C-reactive protein
PRO: Patient-reported outcome
HAQ: Health Assessment Questionnaire
IPQ-R: Revised Illness Perception Questionnaire
SF-36: Short-Form 36
UCL: Utrecht Coping List
PCS: Physical Component Score
MCS: Mental Component Score
QoL: Quality of life
LMM: Linear mixed model
RF: Rheumatoid factor
ACPA: Anti-citrullinated peptide antibodies
