# Correction to: Treatment response and several patient-reported outcomes are early determinants of future self-efficacy in rheumatoid arthritis

**DOI:** 10.1186/s13075-021-02669-7

**Published:** 2021-11-15

**Authors:** Michaël Doumen, Diederik De Cock, Sofia Pazmino, Delphine Bertrand, Johan Joly, René Westhovens, Patrick Verschueren

**Affiliations:** 1 Department of Development and Regeneration, KU Leuven, Skeletal Biology and Engineering Research Centre, ON IV Herestraat 49 - bus 805, 3000 Leuven, Belgium; 2 Rheumatology, University Hospitals Leuven, Leuven, Belgium

**Correction to: Arthritis Res Ther 23, 269 (2021)**

**https://doi.org/10.1186/s13075-021-02651-3**

Following publication of the original article [1], the authors reported an error in the presentation of author names, with first names and surnames being reversed.

The original article [1] has been updated. No changes were made to the article’s content.
