# Supplementary material for: Treatment response and several patient-reported outcomes are early determinants of future self-efficacy in rheumatoid arthritis
Source: Arthritis Res Ther. 2021 Oct 27;23:269. doi: 10.1186/s13075-021-02651-3 (PMC8549201; doi:10.1186/s13075-021-02651-3)
Supplement: Supplementary file 1 — Additional file 1: Supplement 1. Standardised coefficients and bootstrapped CIs for direct and indirect effects of treatment response on self-efficacy. [file 13075_2021_2651_MOESM1_ESM.docx]

**Supplement 1.** Standardised coefficients and bootstrapped CIs for direct and indirect effects of treatment response on self-efficacy.

| **Mediators** | **Coefficient (95% CI)** | **P-value** |
| --- | --- | --- |
| Direct effect | 0.01 (-0.08, 0.11) | 0.79 |
| Indirect: SF-36 MCS | -0.02 (-0.05, 0.01) | 0.13 |
| Indirect: IPQ-R Illness coherence | -0.01 (-0.03, 0.01) | 0.33 |
| Indirect: IPQ-R Emotional representations | -0.03 (-0.07, 0.00) | 0.08 |
| Indirect: IPQ-R Consequences | -0.18 (-0.24, -0.12) | **<0.001** |
| Indirect: IPQ-R Treatment control | -0.01 (-0.04, 0.02) | 0.36 |
| Indirect: IPQ-R Personal control | -0.03 (-0.05, -0.01) | **0.01** |
| Indirect: VAS Pain | -0.03 (-0.10, 0.03) | 0.33 |
| Indirect: VAS Fatigue | -0.04 (-0.08, 0.00) | **0.04** |
| Indirect: HAQ | -0.03 (-0.09, 0.04) | 0.39 |
| Indirect: RAQoL | -0.12 (-0.20, -0.04) | **0.01** |
| Indirect: PGA | -0.04 (-0.10, 0.02) | 0.21 |
| Indirect: SJC28 | -0.01 (-0.02, 0.01) | 0.45 |
| Indirect: CRP | 0.00 (-0.02, 0.01) | 0.94 |
| **Total effect** | **-0.54 (-0.65, -0.43)** | **<0.001** |

Results were obtained from a mediation analysis of the effect of treatment response on total ASES-scores at week 52 and week 104. Reported coefficients for the indirect effects were obtained by multiplying the coefficients within each pathway. Treatment response was treated as an ordinal variable with lower values representing earlier and more persistent response.

*ASES = Arthritis Self-Efficacy Scale, SF-36 = Short-Form 36, MCS = mental component score, IPQ-R = Revised Illness Perception Questionnaire, VAS = Visual Analogue Scale, HAQ = Health Assessment Questionnaire, PGA = patient’s global assessment of disease activity, SJC28 = swollen joint count in 28 joints, CRP = C-reactive protein*
